# Preliminary Study on Fish Scale Collagen Lamellar Matrix as Artificial Cornea

**DOI:** 10.3390/membranes11100737

**Published:** 2021-09-28

**Authors:** Guoping Cheng, Liang Chen, Huanhuan Feng, Bo Jiang, Yi Ding

**Affiliations:** 1 Department of Periodontics, West China College of Stomatology, Sichuan University, Chengdu 610041, China; cgp19940611@foxmail.com (G.C.); clworkzone@126.com (L.C.); 2 State Key Laboratory of Oral Diseases, Sichuan University, Chengdu 610041, China; 3 National Engineering Research Center for Biomaterials, Sichuan University, Chengdu 610041, China; haizhongzhilu@163.com

**Keywords:** corneal stroma, fish scale, collagen lamellar matrix, cell compatibility

## Abstract

To construct a novel artificial cornea biomaterial, a method to prepare collagen lamellar matrix was developed in this study using grass carp scales as raw materials. The relationship between the structure of fish scale collagen lamellar matrix and the optical and mechanical properties was analyzed, and co-culture of it and rat bone marrow mesenchymal stem cells (BMSCs) was performed to preliminarily analyze the cellular compatibility of fish scale collagen lamellar matrix. The results show that the grass carp scales could be divided into base region, lateral region and parietal region according to the surface morphology. The inorganic calcium in the surface layer could be effectively removed by decalcification, and the decalcification rate could reach 99%. After etching treatment, homogeneous collagen lamellar matrix could be obtained. With the decalcification and etching treatment, the water content of the sample increased gradually, but the cross-linking treatment had no obvious effect on the water content of fish scale collagen lamellar matrix. Fish scale collagen lamellar matrix has good transparency, refractive index, mechanical properties and cellular compatibility, which may represent a prospect for the construction of cornea tissue engineering products.

## 1. Introduction

As the main body of human cornea, stroma accounts for 70% of the dry weight of cornea, which is mainly composed of collagen fibers [1]. The transparency of corneal stroma is related to the small diameter of collagen fibers, which are dense and arranged in an orderly manner. Corneal lesions or adverse physiological conditions can change the diameter of collagen fibers or affect the orderly arrangement of fibers, resulting in corneal opacity [2]. The specific complex structure of corneal stroma makes cornea tissue engineering very difficult [3,4]. The ideal tissue-engineered corneal scaffold material requires a wide range of sources, stable physical and chemical properties, high transparency, good air permeability and sufficient mechanical strength, so as to enhance surgical tolerance. Meanwhile, the material requires appropriate curvature and a certain refractive ability, so that the purpose of corrected visual acuity can be achieved after implantation [5]. If the tissue-engineered full-thickness cornea with similar structure and function as cornea can be constructed in vitro, it will be able to solve the serious shortage of donor cornea.

The diameter of collagen fibers in corneal stroma is uniform and fine. The parallel arrangement of collagen fiber sheets is stacked in different directions to form an ordered three-dimensional structure, which provides tensile strength and transparency for the cornea. Currently, the carrier scaffold materials for tissue engineering cornea construction can be divided into natural materials and synthetic materials [6]. The biocompatibility of synthetic materials is poor, and their degradation products may have toxic effects on organisms. Some progress has been made in the construction of tissue-engineered cornea using collagen and other natural biomaterials as scaffolds in vitro. However, the collagen scaffolds prepared in most studies lack highly organized three-dimensional collagen fiber structures, which are crucial for the biomechanical and optical properties of corneal stroma [7]. In recent years, many scholars have used collagen fibers to construct corneal stroma-like scaffolds to bionically design the corneal stromal layer. Wray et al. [8] prepared collagen fibers with uniform and orderly arrangement by electrostatic spinning technology, and successfully cultured rabbit corneal fibroblasts on the fiber membrane, which provides a feasible scaffold material for the construction of tissue-engineered cornea. However, the preparation of collagen fibers with appropriate diameter and arrangement structure and the construction of structural materials with the same thickness as the normal cornea remains to be further studied. Torbet et al. [9] prepared the collagen–proteoglycan membrane with orthogonal lamellar arrangement in a magnetic field, which provides a new idea for the preparation of lamellar structural materials, but whether it can be used in tissue engineering needs further verification in vivo.

The current research results show that in the research of corneal scaffold materials, if only whether the components of materials adapt to cell growth or toxicity is concerned, the optical or mechanical properties of disordered scaffolds can not meet the performance requirements of corneal implants. Based on the lamellar structure of some special connective tissues in animals, can corneal stroma scaffold be constructed by using the similarity of supramolecular structure, texture structure and liquid crystal structure with the target corneal stroma? van Essen et al. [10] used a tilapia scale collagen matrix to prepare artificial cornea, and carried out the co-culture of it and human keratocytes by taking the Ologen^®^ artificial cornea as the control group. The results show that the tilapia scale collagen matrix did not show cytotoxicity, and there was no significant difference in Ologen^®^ inflammatory reaction between the two groups after it was implanted subcutaneously in rats. When it was implanted into rabbit eyes, the rabbit corneal epithelium was healthy, and histological analysis showed no infiltrating inflammatory cells during the 1-year follow-up study, which meets the basic criteria for artificial cornea.

China is a big country with abundant fish and aquatic products in the world. In 2018, total fish production reached 36.971 million tons in China. Grass carp, as one of the four major Chinese carps, produces a lot of by-products, such as fish scales in the processing process, and most of the fish scale resources are wasted [11]. With the deepening understanding of fish scales, it was found that the main component of scales was collagen, with regular lamellar structure. Some studies have shown that fish scale collagen has good biocompatibility, low immunogenicity and low production cost [12].

Therefore, based on the previous research of the research group, this study intends to further analyze the relationship between the structure of collagen lamellar matrix derived from fish scales and optical and mechanical properties, and test the effectiveness of the treatment method, further optimize the preparation process and finally carry out a co-culture of it and rat bone marrow mesenchymal stem cells, to preliminarily analyze the cellular compatibility of fish scale collagen lamellar matrix and verify the methodological feasibility of the idea: “corneal stroma is replaced by collagen lamellar matrix derived from grass carp scales”.

## 2. Materials and Methods

All experiments were conducted according to the ethical protocol and approved guidelines. Ethical approval was obtained from the ethical committees of the West China School of Stomatology, Sichuan University and the State Key Laboratory of Oral Diseases (ethics code WCHSIRB-D-2020-220).

### 2.1. Fish Scale Structure and Composition Analysis

#### 2.1.1. Surface Structure Analysis

Grass carp scales with a diameter of 15–20 mm (Chengdu, China) were selected to observe the surface pattern of scales under a stereoscopic microscope (Olympus, Tokyo, Japan) with a magnification of 75 times. Adobe Photoshop CC 2019 was used to synthesize the image, so as to obtain the complete image of fish scale surface.

#### 2.1.2. Analysis of Calcium Content

The dried fish scales were cut into 3 mm × 3 mm pieces, and a certain mass of crushed fish scales were accurately weighed and soaked in 1 mol/L hydrochloric acid solution for 24 h at room temperature. A total of 1 mL hydrochloric acid decalcification solution was taken and diluted to 20 mL, then 15 mL ammonia buffer (pH = 10) and 2 drops of Eriochrome Black T Indicator (Innochem, Beijing, China) were added to prepare the test solution. Then, according to EDTA (Solarbio, Beijing, China) complexometric titration, the content of calcium ions was determined in the solution to be measured. The mass fraction of calcium ions in fish scale was calculated according to the following Equation (1):

(1) $$\omega_{1}=\frac{\rho 1\times V1\times V2}{m\times V3\times 1000}\times 100\%$$

#### 2.1.3. Analysis of Decalcification Rate

A total of 1 mL original decalcification solution was taken, 19 mL distilled water, 15 mL ammonia buffer (pH = 10) and two drops of Eriochrome Black T Indicator were added, shaken well and standard zinc solution was used for titration. When the indicator color changed from blue to purple, the titration was finished, and the titration volume was recorded as *V*_0_. After decalcification, 1 mL original decalcification solution was taken to record the titration volume *V*_1_ according to the above method. The decalcification rate was calculated according to the following Equation (2):

(2) ω%=(V0−V1)×C×M×V×10−3m×ω1

### 2.2. Preparation of Fish Scale Collagen Lamellar Matrix

Grass carp scales, of about 20 mm diameter and 1000 g weight, were collected (Chengdu, China), and the preparation of fish scale collagen lamellar matrix was based on our previous research [13]. In brief, fresh fish scales were washed successively with distilled water, mixed solvent and buffer at 15 °C to remove mucopolysaccharides, proteins and fats. The scales were soaked in acid-mixed solution (Kelong, Chengdu, China) at 4 °C for decalcification for about 4 h, then washed with water and incubated in acetic acid solution at 4 °C for 1 h, which was called decalcified fish scale. The acid etching solution was sprayed on the surface of decalcified fish scales every hour for etching, etching only one side of the bone layer and not etching the inner layer, which was called fish scale collagen lamellar matrix. Then, the collagen lamellar matrix was cross-linked in an EDC-NHS (Aladdin, Shanghai, China) solution at 4°C for 24 h, which was called cross-linked collagen lamellar matrix. Finally, the above materials were soaked in 75% ethanol (Kelong, Chengdu, China) for 12 h for disinfection and stored in 4 °C aseptic PBS buffer.

### 2.3. Detection for Physical and Chemical Properties of Fish Scale Collagen Lamellar Matrix

#### 2.3.1. Thickness Characterization

The cross-sections of fresh fish scale, decalcified fish scale and fish scale collagen lamellar matrix were scanned by micro-CT (Scanco, Zurich, Switzerland). The voltage was set as 45 kV, the current was set as 177 μA and the scanning resolution was set as 5 μM.

#### 2.3.2. Water Content Analysis

Three pieces of fresh fish scale, decalcified fish scale, fish scale collagen lamellar matrix and cross-linked collagen lamellar matrix were randomly selected and weighed after freeze drying, which was recorded as dry weight, *W*_1_, then soaked in PBS solution for 24 h; the surface moisture was absorbed by filter paper and weighed as wet weight, *W*_2_. The water content of the membrane was calculated according to the following formula:

$$Water\;content=\left(W_{2}-W_{1}\right)∕W_{2}\times 100\%$$

#### 2.3.3. In Vitro Atomization Analysis

Simulated body fluid (SBF, Aladdin, Shanghai, China), simulated tear fluid (STF, Aladdin, Shanghai, China) and 0.1% sodium hyaluronate artificial tear fluid (Kelong, Chengdu, China) were used as atomization solutions, and deionized water as blank control. The deposition of fish scale collagen lamellar matrix and its influence on light transmittance after several days of immersion at 37 °C were investigated. In the experiment, the fish scale collagen lamellar matrix samples were selected, each sample was put into a centrifuge tube and 5 mL solution was added to make the sample completely immersed. The sample was placed in a 37 °C water bath, and the solution was changed once a day. After soaking in different solutions for four weeks, the samples were taken out for electron microscope–energy dispersive spectrum (EDS, JEOL, Tokyo, Japan) analysis, and the light transmittance was detected by ultraviolet-visible spectrophotometer (Hitachi, Tokyo, Japan).

#### 2.3.4. Optical Property Detection

An ultraviolet-visible spectrophotometer was used to measure the transmittance value of samples with different hierarchies in the wavelength range of visible light (400–800 nm) every 1 nm, and the transmittance curve was drawn. The transparency changes of fish scales after treatment were investigated, and the factors affecting the transparency of collagen matrix were analyzed. The refractive index of samples with different hierarchies was measured by IR140 temperature-controlled intelligent refractometer (Insmark, Shanghai, China). The sample was cut to the appropriate size, and the surface moisture was absorbed with filter paper, and then placed on the sample table. Diiodomethane was added to the sample as the protection solution. The temperature was adjusted to 37 °C and the refractive index of the sample was determined. Three samples were randomly taken from each group, and the average value of the results was taken.

#### 2.3.5. Mechanical Property Detection

**Figure 1:**
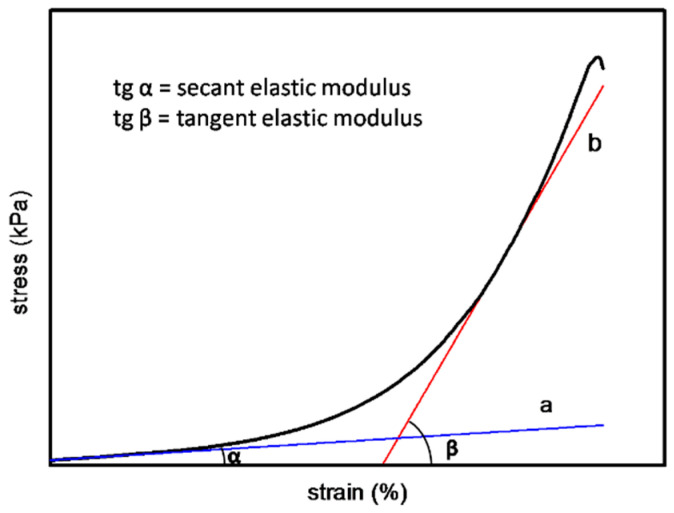
The illustration for secant elastic modulus and tangent elastic modulus.

The humid samples of different hierarchies were cut into regular strips with a certain length and width. The thickness of the samples was measured by a screw micrometer (Diatest, Darmstadt, Germany). The stress–strain curve of the material was nonlinear in the elastic range, which could be represented by secant modulus and tangent modulus. As shown in Figure 1, the slope of tangent A is defined as secant elastic modulus (Es), and tangent B is defined as tangent elastic modulus (Et).

### 2.4. Cellular Compatibility Detection of Fish Scale Collagen Lamellar Matrix

#### 2.4.1. Primary Culture of Rat Bone Marrow Mesenchymal Stem Cells (BMSCs)

Two male SD rats, 3–4 weeks old, SPF grade and weighing 200 g were purchased from Chengdu Dashuo Experimental Animal Center, killed by cervical vertebra dislocation and placed in 75% ethanol solution for 10 min. The bilateral tibia and femur were aseptically separated under sterile conditions. Subsequently, bilateral epiphyses were cut off and 5 mL αMEM (Gibco, New York, NY, USA) medium containing 10% fetal bovine serum (Gibco, New York, NY, USA) and 1% penicillin/streptomycin (Hyclone, Logan, UT, USA) was extracted with a sterile syringe to repeatedly rinse the medullary cavity and collect bone marrow. The collection was centrifuged at 1000 r/min for 5 min, then the supernatant was discarded and inoculated into the culture bottle again. The culture was maintained at 37 °C in a humidified atmosphere of 95% air and 5% CO_2_. After 24 h, the medium was changed for the first time, and then it was changed every two or three days. The cell proliferation and growth status were observed by the inverted phase-contrast microscope. When the culture reached a confluence of 80%, the cells were passaged, and the third generation of bone marrow mesenchymal stem cells was selected for subsequent experiments.

#### 2.4.2. BMSCs Adhesion

Scanning electron microscopy (SEM, Hitachi, Tokyo, Japan) was used to examine the morphological characteristics of BMSCs cultured onto the fish scale collagen lamellar matrix. Fish scale collagen lamellar matrix was trimmed with a punch (12 mm in diameter) and placed onto 24-well culture plates. BMSCs were seeded onto fish scale collagen lamellar matrix which had been placed one piece per well in 24-well plates at the density of 1 × 10^5^ cells/well and cultured for 3 h at 37 °C in a CO_2_ incubator. After 3 h, the culture medium was removed by aspiration, and each well was washed twice with PBS. Then, cells were fixed in 2.5% glutaraldehyde for 12 h at 4 °C. After the fixative was aspirated, 50, 70, 90 and 100% ethanol solution were added to each well for gradient dehydration, each time for 10 min; 50, 70, 90 and 100% isoamyl acetate were added to the plate for gradient dealcoholization, each time for 10 min; and the above samples were dried in a CO_2_ critical point dryer for 1 h. Finally, gold sputtering was carried out, and the samples was observed by SEM.

#### 2.4.3. BMSCs Proliferation

According to ISO 10993-5-2009, eluate of the biomaterials was extracted and was used for a cell compatibility test. Fish scale collagen lamellar matrix was cut into a square with a side length of 7 mm. Every three pieces of fish scale collagen lamellar matrix were placed onto a 24-well plate, and 2.5 mL of αMEM medium was added to each well. After incubation for 24 h, the eluate was extracted and stored at 4 °C for reserve. A CCK-8 kit (Biyuntian, Shanghai, China) was used to detect the effect of fish scale collagen lamellar matrix on cell growth. The third-generation BMSCs were digested with 0.25% trypsin (Hyclone, Logan, UT, USA) and collected according to the above methods. The cells were resuspended with the eluate or complete medium and were seeded on 96-well plates with a density of 1 × 10^4^ cells/mL, with 100 μL in each well. The conventional adherent culture was used as the control group. After 1, 3, 5 and 7 days of culture, a plate was taken and 10 μL CCK-8 was added, which was then incubated in a 5% CO_2_ incubator at 37 °C for 2 h, and the absorbance at 450 nm was determined. The mean value of each well was calculated first, and then the mean ± standard deviation (*n* = 5) of the group was obtained by using the values of 5 parallel samples.

### 2.5. Statistical Analysis

SPSS 20.0 and Origin 8.5 software were used for data analysis. The measurement data that conformed to a normal distribution were expressed as mean ± standard deviation, and the data were compared by independent-sample t-tests or paired-sample t-tests. *p* < 0.05 was regarded as a statistical difference.

## 3. Results

### 3.1. The Result of Fish Scale Structure and Composition Analysis

#### 3.1.1. Surface Structure Analysis of Fish Scale

**Figure 2:**
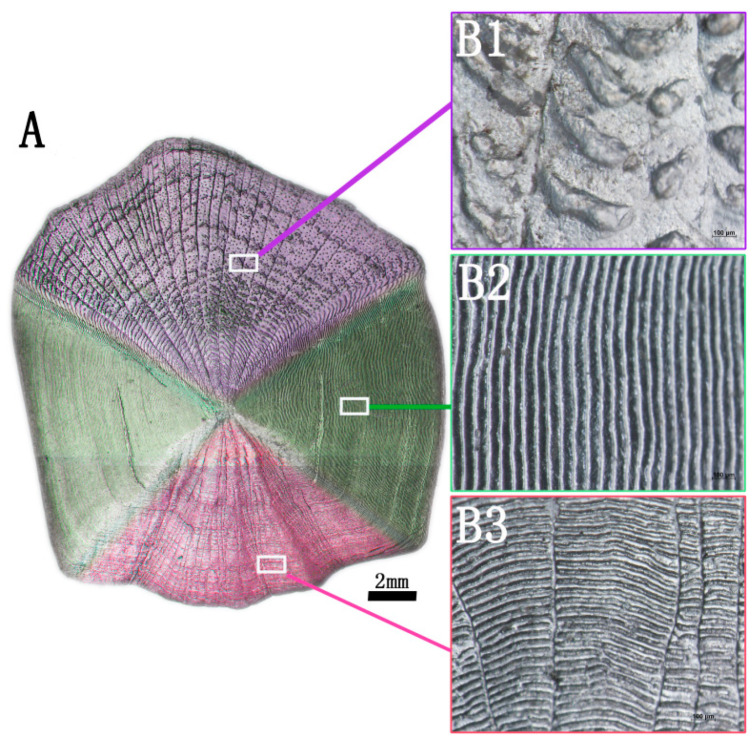
Surface image of grass carp scale (**A**), posterior field of fish scale (**B1**), lateral field of fish scale (**B2**), anterior field of fish scale (**B3**).

The image of the surface of the complete grass carp scale is shown in Figure 2A. There are different micropatterns on the surface of the fish scale. According to the surface morphology, the surface of the fish scale is divided into three parts: the base region with black grain bulge outside (Figure 2B1), the lateral region with regular scale ridge on both sides (Figure 2B2) and the parietal region with a radial scale groove buried under the dermis layer (Figure 2B3). In the subsequent research experiments, the lateral region of the fish scale was taken as the research object.

#### 3.1.2. Analysis of Calcium Content in Fish Scale

**Figure 3:**
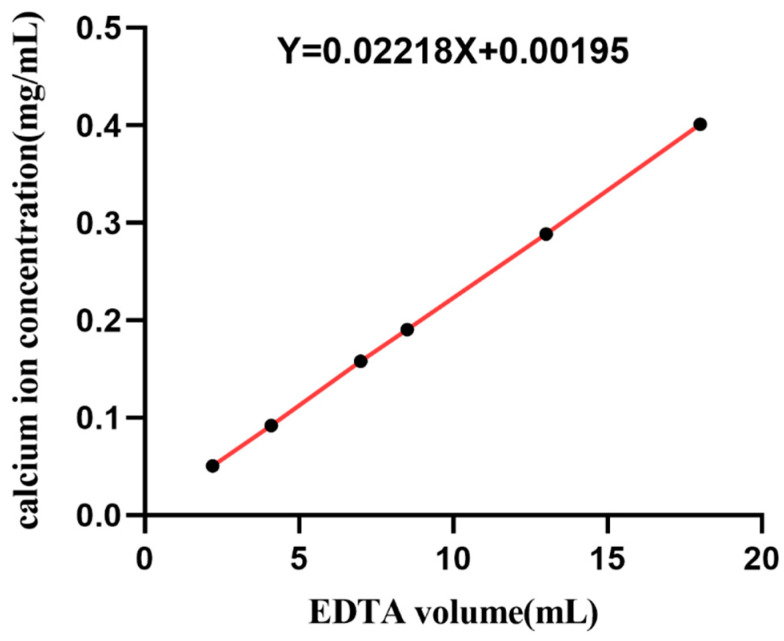
Standard curve of calcium ion concentration.

The standard curve of calcium ion concentration was obtained according to the method in 2.1.2, as shown in Figure 3; the standard curve equation of calcium ion concentration was y = 0.02218x + 0.00195, as shown in Table 1; and the mass fraction of total calcium ions in fish scale was 10.6%.

#### 3.1.3. Analysis of Decalcification Rate

The decalcification rate was calculated according to the method of Section 2.1.3. The results are shown in Table 2, and the average decalcification rate was 99%.

### 3.2. Physical and Chemical Properties of Fish Scale Collagen Lamellar Matrix

#### 3.2.1. Thickness Characterization

**Figure 4:**
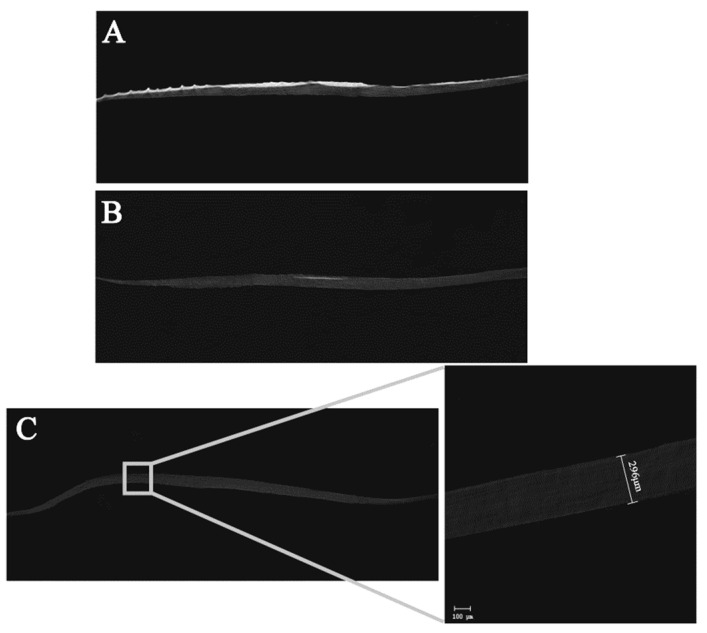
Results of micro-CT. Fresh fish scale (**A**), decalcified fish scale (**B**), fish scale collagen lamellar matrix (**C**).

The cross-section scanning results of samples with different hierarchies by micro-CT are shown in Figure 4. Figure 4A shows a cross-sectional view of fresh fish scales, in which the surface part is highlighted and the bottom layer is gray shadow. It can also be observed that the thickness of fish scales is uneven, being thick in the middle part and thin in the peripheral part. The micro-CT results of decalcified fish scales (Figure 4B) show that only a small white light region appears in the middle of the material surface layer. In the micro-CT image of fish scale collagen lamellar matrix (Figure 4C), there is no highlight part, and the whole material is shown in grey. The thickness of the matrix is not uniform, the thickness in the middle is thick and the periphery is thin—about 150–300 μM. Fish scale collagen lamellar matrix did not keep the original straight shape of the fish scale, and its periphery curved to the inside, showing a curved surface.

#### 3.2.2. Water Content Analysis

**Figure 5:**
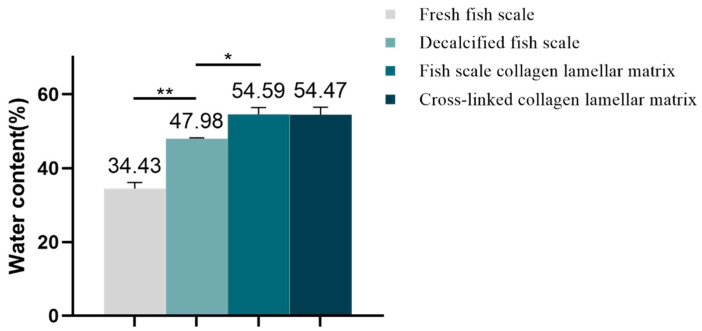
Water content of different samples (*n* = 3). * *p* < 0.05; ** *p* < 0.01.

According to the calculation, the water content of each sample is shown in Figure 5. The water content of fresh fish scale, decalcified fish scale, fish scale collagen lamellar matrix and cross-linked collagen lamellar matrix were 34.43 ± 1.66, 47.98 ± 0.24, 54.59 ± 1.78 and 54.47 ± 2.02%, respectively. With the decalcification and etching treatment, the water content of the sample increased gradually, but the cross-linking treatment had no significant effect on the water content of fish scale collagen lamellar matrix.

#### 3.2.3. In Vitro Atomization Analysis

**Figure 6:**
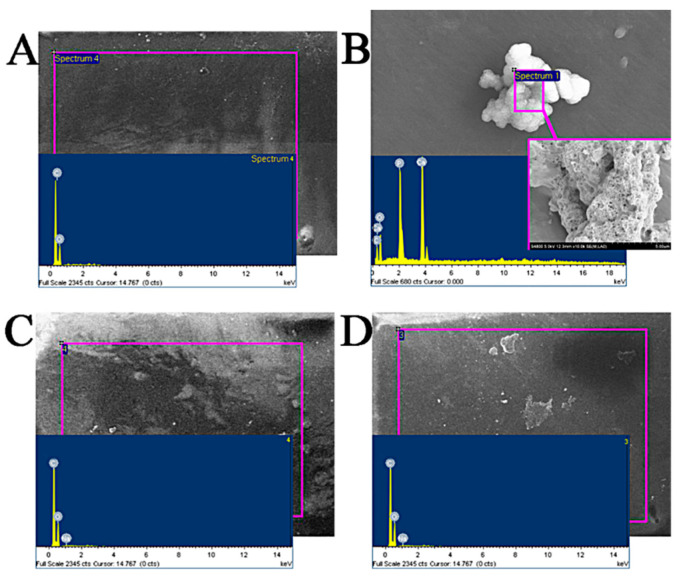
The results of EDS. (**A**) Deionized water group; (**B**) SBF group; (**C**) STF group; (**D**) artificial tears group.

The fish scale collagen lamellar matrix was immersed in different atomization solutions to simulate the change in transparency after implantation. In this experiment, the simulated body fluid was prepared according to Kokubo [14]; the simulated tear fluid (STF) was prepared according to the literature. Fish scale collagen lamellar matrix was immersed in the prepared solutions, and the samples were taken out after 4 weeks. The surface of each sample was gently washed with distilled water and dried. Then, the samples were pasted on the sample table with conductive adhesive for electron microscope–energy dispersive spectrum (EDS) analysis. The EDS analysis atlas is shown in Figure 5, and the data of each element content analyzed by EDS are shown in Table 3. As shown in Figure 6, the EDS results of the deionized water control group show that the material surface only contains C and O elements, and no inorganic phase elements, so other elements in the experimental group are deposited from external solution. After being immersed in SBF for 4 weeks, crystals were formed on the surface of fish scale collagen lamellar matrix. The EDS results show that it should be hydroxyapatite-like crystal with a Ca/P ratio of about 1.48. In the STF group and sodium hyaluronate artificial tear fluid group, Ca and P were not detected on the surface by EDS analysis.

#### 3.2.4. Light Transmittance Analysis

**Figure 7:**
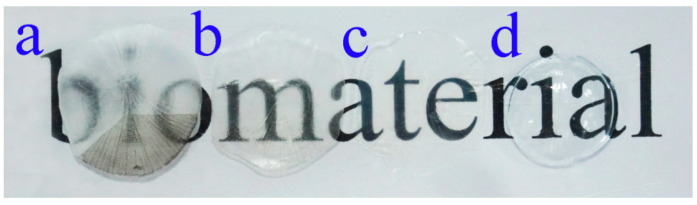
Digital photos of fresh fish scales (**a**), decalcified fish scales (**b**), fish scale collagen lamellar matrix (**c**) and contact lens (**d**).

**Figure 8:**
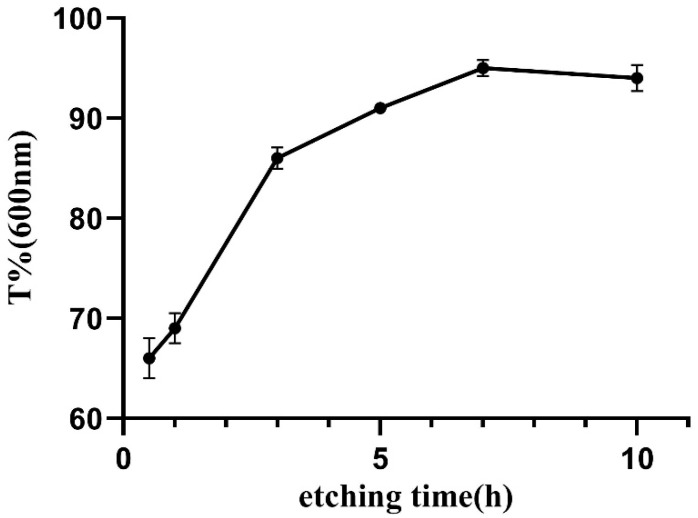
The change in material transmittance at 600 nm with the increase in etching time.

**Figure 9:**
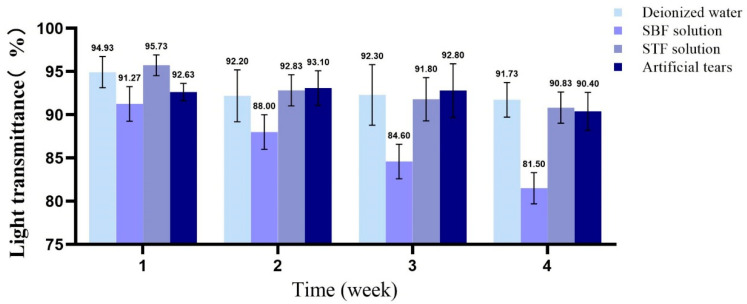
The change in transmittance of fish scale collagen lamellar matrix soaking in different solutions for 4 weeks (*n* = 3).

Taking a contact lens (Haichang, center thickness of 0.08 mm) as the control, the change in the light transmittance of the samples was investigated. Figure 7 shows the digital photos of fresh fish scale, decalcified fish scale, fish scale collagen lamellar matrix and contact lens. With the decalcification and etching treatment, it can be seen that the transparency of the sample is gradually improved, and the transparency of the collagen matrix is seemly equivalent to that of the contact lens. Figure 8 shows that with the increase in etching time, the light transmittance of the material gradually increases, and the rate of increase is faster within 0–3 h. This process mainly removes the residual inorganic phase on the surface. After etching for 7 h, the light transmittance of the material is about 95%. However, further etching will not lead to a significant increase in transmittance. The transmittance of each sample at the wavelength of 600 nm was measured by ultraviolet-visible spectrophotometer (Figure 9). Three parallel samples were set for each group of samples, and the mean value of the measured results was taken. The results show that the average transmittance of the STF and sodium hyaluronate artificial tear fluid groups was more than 90% after 4 weeks of storage at 37 °C. SBF solution had a great influence on the transmittance of the sample, and the transparency decreased with the increase in soaking time.

#### 3.2.5. Refractive Index Analysis

When the light transmitsfrom one medium into another, the propagation speed of the light will change due to the different densities of the two media, and the phenomenon of refraction will occur. The refractive index of samples with different hierarchies was measured by a refractometer. The results are shown in Table 4. With the decalcification and etching treatment, the refractive index of the material decreased, but the cross-linking treatment had no obvious effect on the refractive index. The refractive indices of fresh fish scale, decalcified fish scale, fish scale collagen lamellar matrix and cross-linked collagen lamellar matrix were 1.5887 ± 0.1076, 1.5890 ± 0.0055, 1.3363 ± 0.0054 and 1.3354 ± 0.0060, respectively. The refractive index of fresh fish scales varies greatly among different samples, which may be due to the influence of various environmental factors on the natural scales. The difference between the samples of fish scale collagen lamellar matrix was small, which indicates that the treatment process can ensure that each sample is treated uniformly.

#### 3.2.6. Mechanical Property Detection

**Figure 10:**
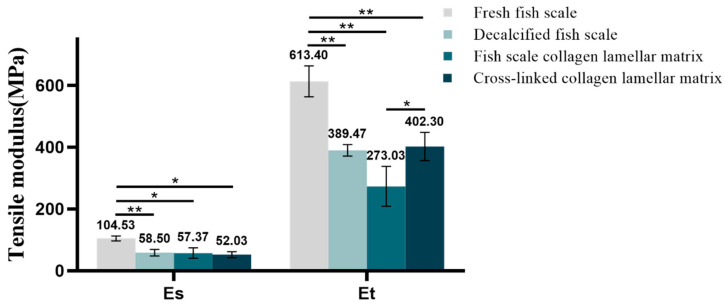
Tensile modulus for different samples (*n* = 3). * *p* < 0.05; ** *p* < 0.01.

**Figure 11:**
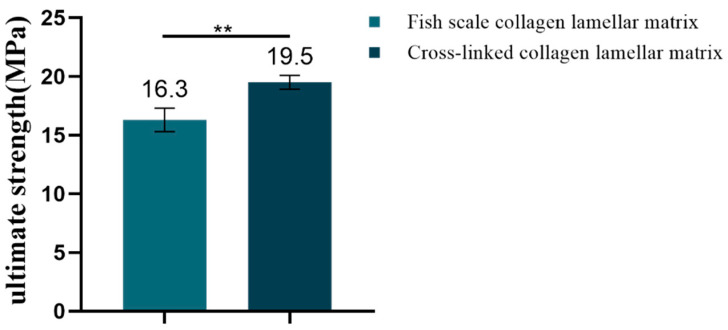
The ultimate strength of fish scale collagen lamellar matrix and cross-linked collagen lamellar matrix (*n* = 3). ** *p* < 0.01.

The Es and Et reflect the strength of the material in the low-strain and high-strain region. The tensile stress–strain curves of samples with different hierarchies are shown in Figure 10. According to the definition of Es and Et, the corresponding data of each sample were calculated by origin software. The Es was 104.53 ± 7.8 MPa for fresh fish scale, 58.5 ± 10.66 MPa for decalcified fish scale, 57.37 ± 17 Mpa for fish scale collagen lamellar matrix and 52.03 ± 9.75 MPa for cross-linked collagen lamellar matrix. The Et was 613.4 ± 50.18, 389.47 ± 18.8, 273.03 ± 64.64 and 402.3 ± 45.43 MPa, respectively. The fracture tensile strength of fish scale collagen lamellar matrix and cross-linked collagen lamellar matrix were 16.3 ± 1.0 and 19.5 ± 0.6 MPa, respectively, as shown in Figure 11.

### 3.3. Cellular Compatibility Test of Fish Scale Collagen Lamellar Matrix

#### 3.3.1. Primary Culture of Rat BMSCs

**Figure 12:**
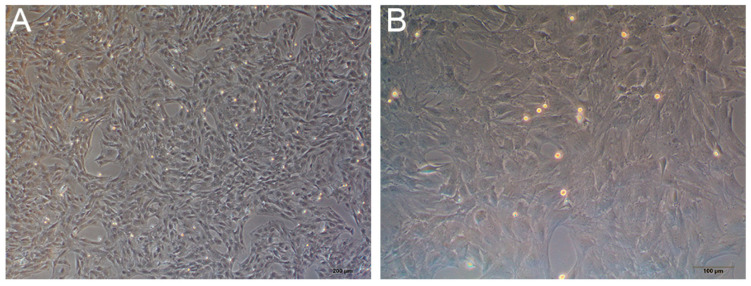
The third generation of primary culture rat BMSCs. (**A**) 40×. (**B**) 100×.

An inverted phase-contrast microscope was used to observe the third generation of rat BMSCs (Figure 12A,B). Cells were grown as adherent monolayers and were spindle or polygon shaped and radially arranged.

#### 3.3.2. Cell Adhesion

**Figure 13:**
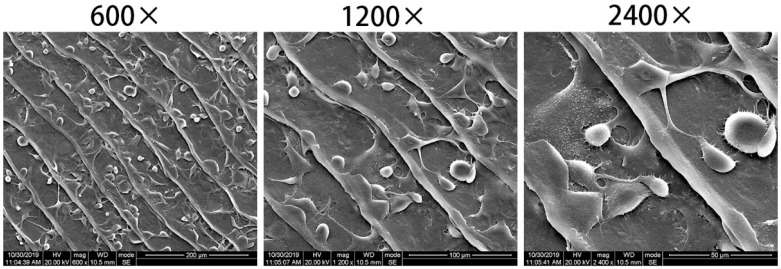
BMSCs adhered to the surface of fish scale collagen lamellar matrix after 3 h of cultivation.

After cell inoculation for 3 h, most of the cells on the surface of the collagen matrix extended slender pseudopodia, and the cells spread fully (Figure 13), indicating that BMSCs adhered well to fish scale collagen lamellar matrix.

#### 3.3.3. Cell Proliferation

**Figure 14:**
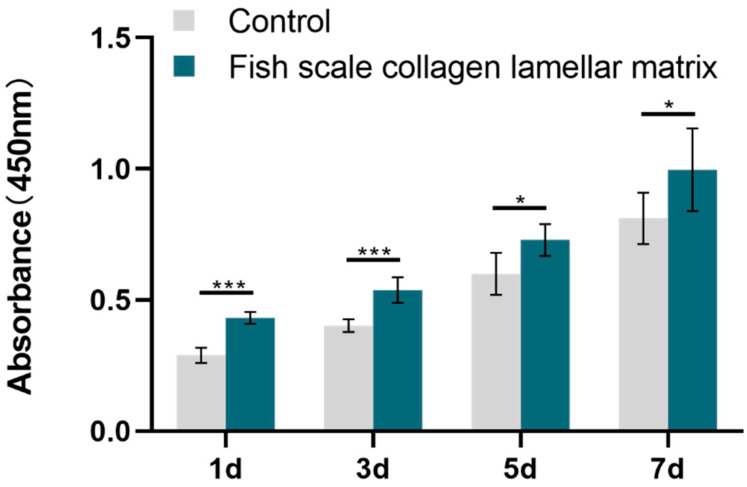
BMSCs proliferation test. * *p* < 0.05; *** *p* < 0.001.

The CCK-8 method showed the effect of eluate on cell proliferation. The number of fish scale collagen lamellar matrix groups at 1, 3, 5 and 7 days was more than that of the conventional adherent culture group, indicating that fish scale collagen lamellar matrix promoted the proliferation of BMSCs (Figure 14).

## 4. Discussion

In biological connective tissue such as fish scale, there is a normal lamellar structure based on collagen [15], and its fine structure causes the specific tissue to have the corresponding function [16]. The size of fish scales is associated with the type and age of fish. The fish scales used in our study were all from one-year-old grass carp, and we plan to use larger ones from specific fish merchants instead of vegetable markets. Inspired by the decellularization and decalcification methods in the literature [17,18], the acid decalcification solution was used to remove the inorganic components represented by calcium in fish scales, so as to obtain a standard collagen matrix with application value. The content of calcium ion in fresh fish scales was measured by EDTA titration; the residual calcium content in the prepared collagen substrate was about 1%, and the decalcification rate was 99%, indicating that this decalcification method is very effective and can be used for subsequent studies.

There are many methods to improve the stability of collagen, such as glutaraldehyde cross-linking, EDC/NHS cross-linking, dehydrogenation heat treatment, etc. [19]. Among them, EDC/NHS cross-linking is widely used because of its low-dose, non-toxic nature, which can improve the stability of scaffolds, reduce the degradation rate in vivo and has good mechanical properties. In this research, the preparation of cross-linked collagen lamellar matrix was cross-linked with EDC. The water content of the samples with different treatments showed that the water content of the samples increased gradually with the decalcification and etching treatment, indicating that the matrix layer has better water absorption. The water content of materials may be affected by cross-linking [20]. However, in this study, the fibers in the matrix layer were closely arranged and the collagen lamellar matrix itself had a high degree of cross-linking, so the water content of fish scale collagen lamellar matrix exhibited no significant change after cross-linking treatment.

In clinical and animal implantation experiments, calcium deposition occurs in many collagen-based materials after implantation, resulting in reduced transparency and affecting visual acuity [10]. The SBF immersion test is often used to study the biological activity of materials in vitro. Calcium deposition was detected on the surface of fish scale collagen lamellar matrix after soaking in SBF solution for 4 weeks, indicating that the material has good biological activity. The composition of the SBF solution is quite different from that of tears, which cannot reflect the actual situation of implantation. STF is similar to tears. Sodium hyaluronate is a bioactive substance in the human body and is also distributed in the eye. Its primary characteristic is that it can form a regular, stable and long-acting water film on the surface of the eye, which is not easy to wash away. Therefore, the atomization test in vitro with these two solutions can better reflect the situation of the implanted materials. In the experiment, calcium and phosphorus elements were not detected on the surface of fish scale collagen lamellar matrix after 4 weeks of immersion in these two solutions, which had little effect on the light transmittance of the samples. After immersion for 4 weeks, the light transmittance of the materials could be maintained above 90%, while the transmittance in the control group decreased to a certain extent after 4 weeks of immersion. It shows that the external deposition is not the main factor affecting the light transmittance, which may result from the fact that in the collagen molecules, after being kept at 37 °C for a long time, stability was destroyed.

The results of light transmittance show that decalcification treatment increased the transmittance of fish scale samples by 30–40%. Combined with the analysis of fish scale grading structure, it was indicated that the surface bone layer was the main reason for the affected light transmittance. On the one hand, due to the large surface roughness of fish scales, they possess a strong ability to reflect light and reduce the transmission of light; on the other hand, the refractive index of crystalline hydroxyapatite and amorphous collagen fiber is different, which will cause light diffusion on the interface, resulting in poor transparency. With the gradual erosion of the bone layer and transition layer of fish scale, the light transmittance of the material increased significantly. After etching treatment for 7 h, the light transmittance did not increase. Therefore, etching for 7 h can be regarded as the best etching time. The high transparency of fish scale collagen lamellar matrix depends on uniform fiber size and ordered lamellar arrangement. However, the cross-linking treatment had a certain effect on the transparency of the material, but the collagen matrix still maintained a high light transmittance, which was about 93% in the visible light range, which is similar to that of human cornea [21,22].

Cornea, as one of the refractive system tissues of the eyeball, has a certain refractive index, which creates the clear image on the retina of external objects. The refractive index of the human cornea is 1.376, and the refractive index of fish scale collagen lamellar matrix is between 1.336 and 1.337, as the refractive index of decalcified etching material approaches that of the human cornea. The difference in refractive index of samples with different hierarchies may be caused by the mineralization degree of fish scales from top to bottom. In the mineralization process of fish scales, the surface layer of fish scale is the first mineralized layer, which is the initial calcification structure of the fish scale, and the initial calcification site is matrix vesicles, which contain rods that grow with matrix to form a fully mineralized outer layer [23]. With the formation of organic matrix, the mineralization process proceeds rapidly, and the outer hydroxyapatite crystal enters the inner collagen fiber space [24]. In the stromal layer of some fish scales, some fine fibers were found perpendicular to the lamellar direction and embedded in the collagen lamellae. It seems that mineralization started from these vertical fibers [25]. Therefore, the mineralization degree of different lamellar regions of the matrix is different, and the refractive indexes of two surfaces of the same matrix are also different, which is also based on this reason.

Corneal scaffold materials need to have certain mechanical properties to withstand surgical suture. In addition, studies have shown that good mechanical strength of scaffold materials has a certain supporting effect on the cell growth environment and an auxiliary effect on the structure and function of new tissues [26]. With the decalcification and etching treatment, the inorganic phase in the surface layer of the fish scale was gradually removed. Both the Es and Et decreased, indicating that the hard bone layer on the surface of the fish scale was the main reason for the strong anti-deformation ability of the fish scale. The results show that fish scale collagen lamellar matrix still maintains good toughness and strength, which results from the highly ordered lamellar structure of collagen matrix and the organic combination of collagen fibers and hydroxyapatite. Cross-linking treatment can induce the formation of new covalent bonds between collagen molecules, thus effectively improving the mechanical properties of the materials. At present, there are few reports in the literature on the mechanical behavior of collagen laminate matrix. Therefore, the comparison of the mechanical properties of matrix layers with different plywood structures may be an interesting study.

Tissue engineering technology is used to construct artificial cornea, and scaffolds as a carrier of cell adhesion, growth and differentiation must have good cell and tissue compatibility to support cell growth in vitro or in vivo. Rat BMSCs have active proliferation and multi-directional differentiation potential. In addition, they can be easily obtained, extracted, cultured and expanded [27,28]. Meanwhile, they are commonly used in in vitro experimental cells of tissue engineering and are also used to test the biocompatibility of materials [29]. In the cell adhesion test, scanning electron microscopy showed that BMSCs adhered and spread well on the surface of fish scale collagen lamellar matrix; in the cell proliferation experiment, the proliferation of BMSCs of the fish scale collagen lamellar matrix group was better than that of conventional adherent culture; the results show that fish scale collagen lamellar matrix had good cellular compatibility.

Due to the particularity of corneal tissue, there are many high requirements for the transparency and mechanical strength of scaffold materials. In many studies, collagen hydrogels or collagen sponges based on collagen did not achieve the ideal requirements for transparency and intensity of artificial corneas. The main reason was that the membrane materials prepared could not achieve the same high and regular arrangement in structure as corneal stroma [19,30]. The fish scale collagen lamellar matrix prepared in this study has a natural collagen lamellar structure, which shows great advantages in optical and mechanical properties. However, more studies on safety and effectiveness are needed for the construction of cornea in vitro, which needs to be optimized in many aspects; for example, how to achieve uniform swelling of fish scale collagen lamellar matrix, how to use fish scale collagen lamellar matrix to construct functional full-thickness cornea, etc.

## 5. Conclusions

According to the surface morphology of a grass carp scale, it can be divided into three parts: base region, lateral region and parietal region. The surface inorganic calcium can be effectively removed by decalcification, and the decalcification rate can reach 99%. After etching treatment, homogeneous collagen matrix can be obtained. With the decalcification and etching treatment, the water content of the sample increased gradually, but the cross-linking treatment had no obvious effect on the water content of collagen matrix. Fish scale collagen lamellar matrix has good transparency, refractive index, mechanical properties and cellular compatibility, which may bring hope for the construction of cornea tissue-engineered products.

## Figures and Tables

**Table 1:** Determination and calculation results of calcium content in fish scales.

	EDTA Consumption Volume (ML)	Mass Concentration of Calcium Ion in Decalcification Solution (mg/mL)	Calcium Content in Fish Scale (%) (Mean)
Sample 1	9.2	0.206	10.6
Sample 2	9.8	0.219	
Sample 3	9.6	0.215	

**Table 2:** Determination and calculation results of decalcification rate.

	Volume of Standard Zinc Consumed in Titration of Original Decalcification Solution *V*_0_ (mL)	Standard Zinc Volume Consumed by Decalcification Solution after Titration and Decalcification *V*_1_ (mL)	Decalcification Rate (%) (Mean)
Sample 1	5.0	2.9	99
Sample 2	5.1	2.9	
Sample 3	5.0	3.0	

**Table 3:** The data of element content by EDS.

Element		C	O	Na	Ca	P
Percentage of atomic content (%)	Deionized water group	67.59	32.41	--	--	--
	SBF group	35.77	45.62	--	7.49	11.12
	STF group	66.8	32.99	0.21	--	--
	Artificial ear fluid group	61.96	37.3	0.74	--	--

**Table 4:** Refractive index of different samples.

	Refractive Index			
Sample	Fresh Fish Scale	Decalcified Fish Scale	Fish Scale Collagen Lamellar Matrix	Cross-Linked Collagen Lamellar Matrix
1	1.6566	1.5944	1.3314	1.3285
2	1.6449	1.5835	1.3421	1.3386
3	1.4646	1.5890	1.3353	1.339
Mean (SD)	1.5887 (0.1076)	1.5890 (0.0055)	1.3363 (0.0054)	1.3354 (0.0060)

## Data Availability

Not applicable.
